# Artificial intelligence to detect noise events in remote monitoring data

**DOI:** 10.1002/joa3.13037

**Published:** 2024-04-11

**Authors:** Nobuhiro Nishii, Kensuke Baba, Ken'ichi Morooka, Haruto Shirae, Tomofumi Mizuno, Takuro Masuda, Akira Ueoka, Saori Asada, Masakazu Miyamoto, Kentaro Ejiri, Satoshi Kawada, Koji Nakagawa, Kazufumi Nakamura, Hiroshi Morita, Shinsuke Yuasa

**Affiliations:** ^1^ Department of Cardiovascular Therapeutics Okayama University Graduate School of Medicine, Dentistry, and Pharmaceutical Sciences Okayama Japan; ^2^ Cyber‐Physical Engineering Informatics Research Core Okayama University Okayama Japan; ^3^ Division of Industrial Innovation Sciences, Graduate School of Natural Science and Technology Okayama University Okayama Japan; ^4^ Department of Cardiovascular Medicine Okayama University Graduate School of Medicine, Dentistry, and Pharmaceutical Sciences Okayama Japan

**Keywords:** artificial intelligence, five‐fold cross‐validation, intracardiac electrogram, noise event, remote monitoring

## Abstract

**Background:**

Remote monitoring (RM) of cardiac implantable electrical devices (CIEDs) can detect various events early. However, the diagnostic ability of CIEDs has not been sufficient, especially for lead failure. The first notification of lead failure was almost noise events, which were detected as arrhythmia by the CIED. A human must analyze the intracardiac electrogram to accurately detect lead failure. However, the number of arrhythmic events is too large for human analysis. Artificial intelligence (AI) seems to be helpful in the early and accurate detection of lead failure before human analysis.

**Objective:**

To test whether a neural network can be trained to precisely identify noise events in the intracardiac electrogram of RM data.

**Methods:**

We analyzed 21 918 RM data consisting of 12 925 and 1884 Medtronic and Boston Scientific data, respectively. Among these, 153 and 52 Medtronic and Boston Scientific data, respectively, were diagnosed as noise events by human analysis. In Medtronic, 306 events, including 153 noise events and randomly selected 153 out of 12 692 nonnoise events, were analyzed in a five‐fold cross‐validation with a convolutional neural network. The Boston Scientific data were analyzed similarly.

**Results:**

The precision rate, recall rate, F1 score, accuracy rate, and the area under the curve were 85.8 ± 4.0%, 91.6 ± 6.7%, 88.4 ± 2.0%, 88.0 ± 2.0%, and 0.958 ± 0.021 in Medtronic and 88.4 ± 12.8%, 81.0 ± 9.3%, 84.1 ± 8.3%, 84.2 ± 8.3% and 0.928 ± 0.041 in Boston Scientific. Five‐fold cross‐validation with a weighted loss function could increase the recall rate.

**Conclusions:**

AI can accurately detect noise events. AI analysis may be helpful for detecting lead failure events early and accurately.

## Graphical abstract

A convolutional neural network with a pre‐trained model was used to predict noise or nonnoise events. In the analysis of five‐fold cross‐validation with weighted loss function, the trained artificial intelligence (AI) algorithm could predict noise events in intracardiac electrograms with high diagnostic performance. AI could focus on noise.

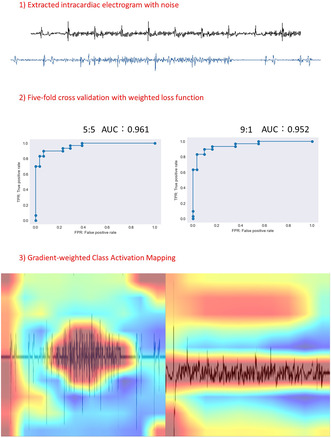

## 1 INTRODUCTION

Cardiovascular implantable electronic devices (CIEDs) have expanded in number and complexity.^1^ Standard ambulatory follow‐up is time‐consuming, and asymptomatic CIED malfunction is difficult to detect in the early stages. Remote monitoring (RM) of CIEDs is advocated as a new standard of care for patients with CIEDs. Several large prospective randomized trials have demonstrated the safety, feasibility, efficacy, and survival improvement of RM. Furthermore, RM has allowed early detection of adverse clinical events, such as arrhythmia, lead failure, and battery depletion.^2^, ^3^, ^4^, ^5^

In some cases, noise events were observed during the analysis of RM data. The causes of noise events were lead failure, electromagnetic interference (EMI), loose set screws, myopotential, or sometimes unknown. It is important to detect noise events early. Lead failure has resulted in life‐threatening events, especially in patients with cardiac pacing dependence, clinical lethal arrhythmia, and high‐voltage implantable cardioverter defibrillator (ICD) leads.^6^, ^7^, ^8^, ^9^, ^10^ RM can detect lead failure earlier,^11^, ^12^, ^13^ which may result in the reduction of inappropriate ICD shocks.^14^, ^15^ However, lead failure is often noted only by arrhythmic events and not by impedance abnormalities.^14^, ^16^ In such cases, human analysis of intracardiac electrograms of arrhythmic events is needed to identify lead failure. In our previous study, 32 (76.2%) of 42 lead failure events were detected as only arrhythmic events.^17^ Noise events caused by EMI, loose set screws, or myopotential are rare, but can also lead to life‐threatening events. In RM data, the number of arrhythmic events is huge,^18^ and it seems impossible to precisely analyze all arrhythmic events by human analysis alone.

Recently, artificial intelligence (AI) and machine learning (ML) have become areas of intense exploration in medicine, showing potential to automate human tasks and even perform tasks beyond human capabilities. For example, AI can predict atrial fibrillation,^19^ left ventricular dysfunction,^20^, ^21^ and hypertrophic cardiomyopathy from a 12‐lead electrocardiogram of sinus rhythm. However, an analysis of the intracardiac electrogram has not yet been reported.

We hypothesized that we could train a neural network to identify noise events in the intracardiac electrogram of RM data. To test this hypothesis, we trained and tested a deep neural network using a large cohort of RM data from Okayama University and its associated hospitals.

## 2 METHODS

### 2.1 Patients followed by RM

This was a retrospective, multicenter study. Since April 2009, patients with CIEDs at Okayama University Hospital and nine associated hospitals have been followed up by the RM center at Okayama University Hospital. A pacemaker (PM), ICD, cardiac resynchronization therapy (CRTP), or CRT with defibrillator (CRTD) was implanted in these patients. RM systems were based on periodic remote follow‐ups plus automatic alerts (Medtronic CareLink [MCL], Minneapolis, MN; Boston Scientific Latitude [BSL], St. Paul, MN). A wired or wireless RM system was used for all patients. The periodic transmission schedules differed (1–4 months) among hospitals. All patients provided written informed consent for the use of the RM system, and the study protocol was approved by the Institutional Review Board and/or Medical Ethics Committee of each hospital.

### 2.2 Analysis of transmitted data and event definitions

All transmitted data were analyzed and summarized in a report by medical engineers and doctors at the RM center (Okayama University Hospital) every working day. If noise events in the intracardiac electrogram were detected, we called the patients and asked them to visit the outpatient clinic or called the attending doctors in the associated hospital. The causes of the noise events were lead failure, EMI. The noise events were defined as the events with short cycle length less than 100 ms or nonphysiological signals. Nonnoise events included real‐time intracardiac electrograms with no arrhythmia, atrial tachyarrhythmia, or ventricular tachyarrhythmia.

### 2.3 AI analysis

**FIGURE 1:**
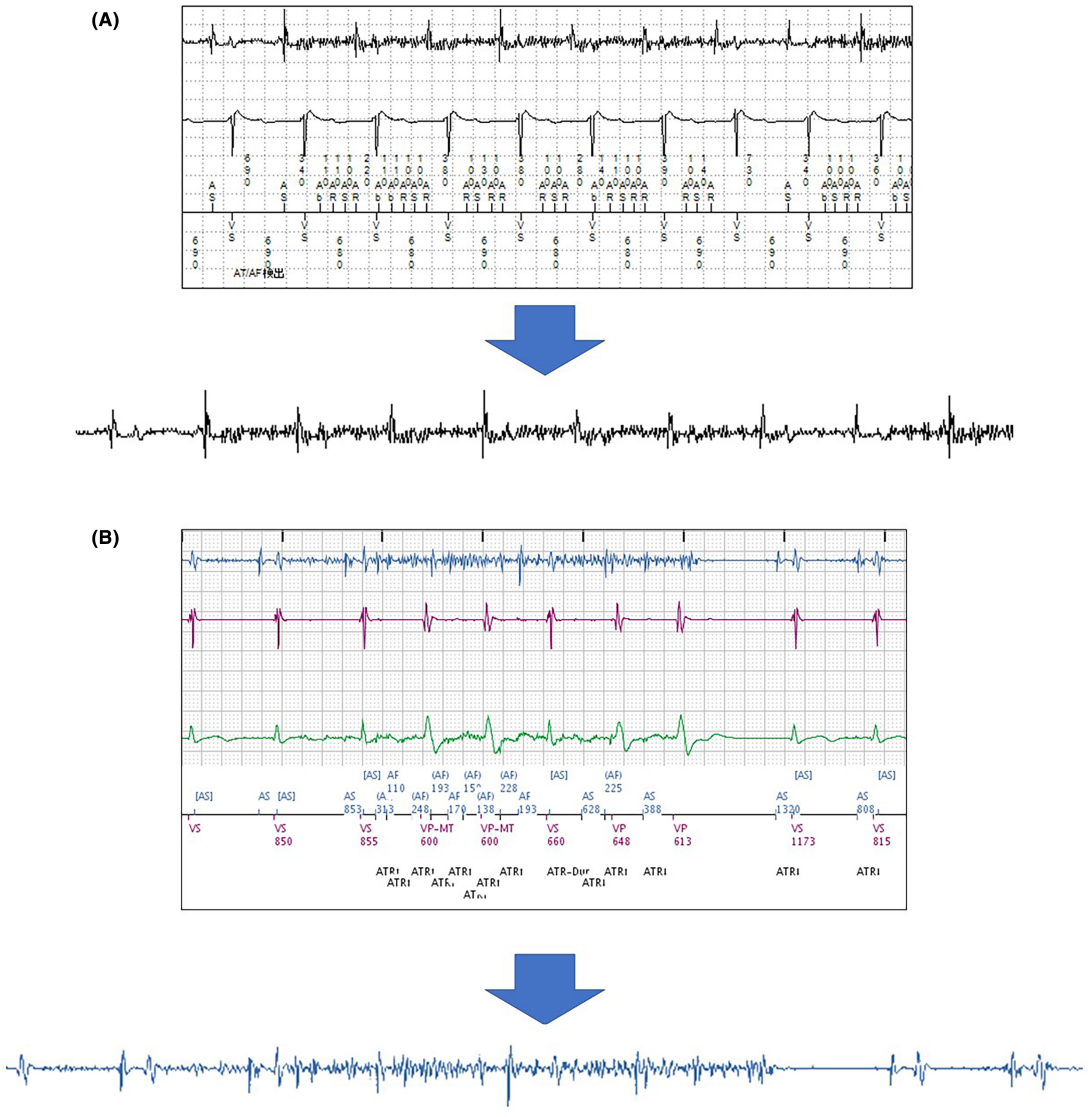
Extraction method for one intracardiac electrogram waveform. (A) Medtronic data: First, an intracardiac electrogram was extracted from the remote monitoring data. Next, only one intracardiac electrogram waveform was extracted. (B) Boston Scientific data.

First, only intracardiac electrogram data were extracted from the RM data, which was not digital but PDF file. In this study, intracardiac electrogram data were recorded from true bipolar atrial lead, true bipolar ventricular lead, and true bipolar ICD lead. Next, these data were assigned to noise and nonnoise events by two expert electrophysiologists. All but pixel values of intracardiac electrogram waveforms were deleted by Python, then, only one intracardiac electrogram waveform with bipolar was extracted from noise and nonnoise events (Figure 1A,B). The waveforms were compressed and resized to 300 × 300 pixel before AI analysis to allow the AI to analyze uniform data.

We analyzed 14 809 RM data points consisting of 12 925 and 1884 Medtronic and Boston Scientific data points, respectively. Among these, 153 (120 events were lead failure, 33 events were EMI) Medtronic and 52 (45 events were lead failure, 7 events were EMI) Boston Scientific data points, respectively, were diagnosed as noise events by two expert electrophysiologists. The data used in the analysis did not include data taken from the same patient. A convolutional neural network (CNN),^22^ which is a hierarchical neural network consisting of a convolution layer and a pooling layer, with a pre‐trained model was used to predict noise or nonnoise events.

The expression of intracardiac electrograms was too different among the companies to analyze the noise events from multiple manufacturers simultaneously. Therefore, an analysis of each manufacturer was necessary.

**FIGURE 2:**
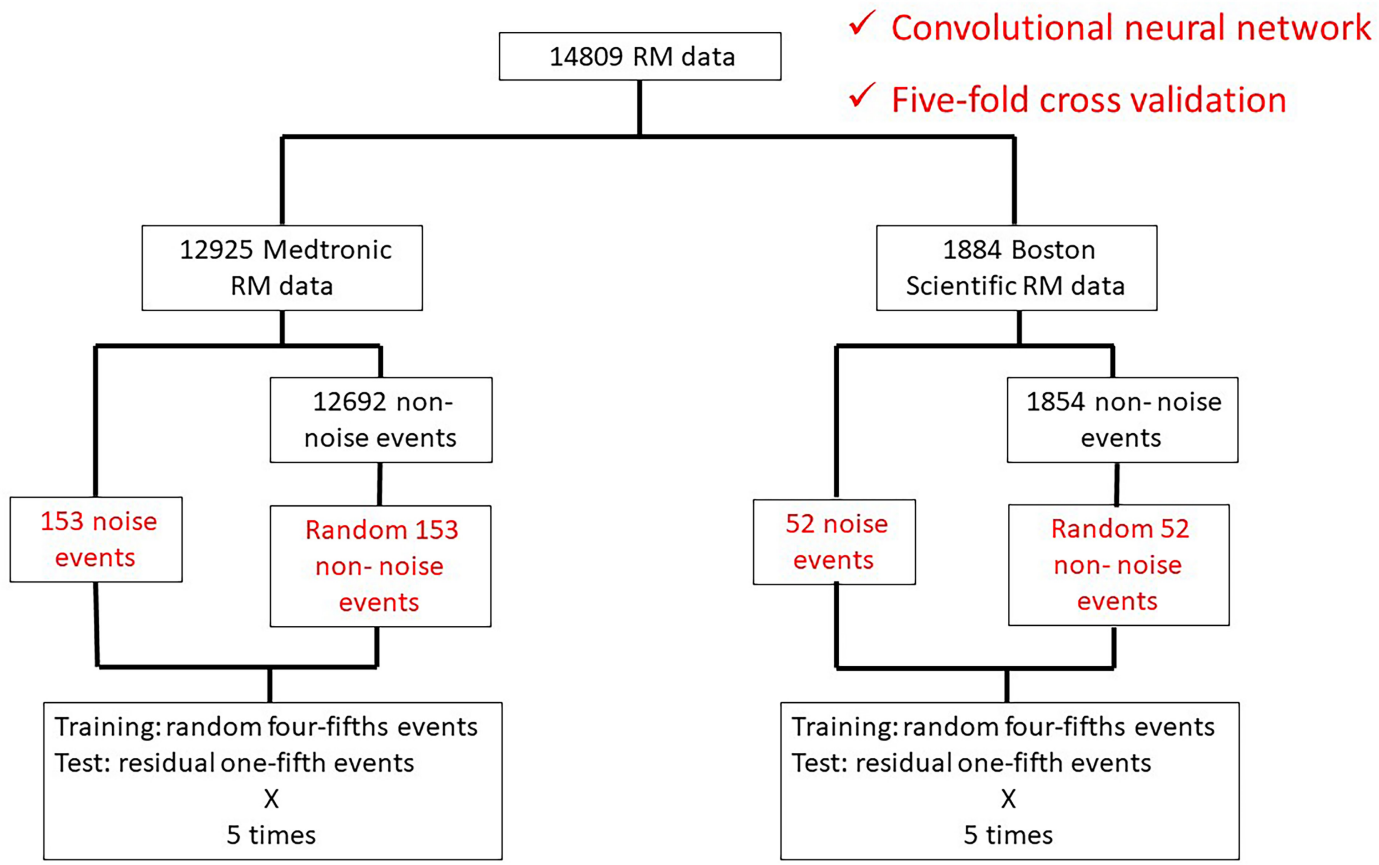
Analyzed remote monitoring (RM) data. We analyzed 14 809 RM data points consisting of 12 925 and 1884 Medtronic and Boston Scientific data points, respectively. Among these, 153 and 52 Medtronic and Boston Scientific data points, respectively, were diagnosed as noise events by human analysis. A convolutional neural network with a pre‐trained model was used to predict noise or nonnoise events. In Medtronic data, 306 events, including 153 noise events and randomly selected 153 out of 12 692 nonnoise events, were used in the five‐fold cross‐validation. The Boston Scientific RM data were analyzed similarly.

In Medtronic data, 306 events, including 153 noise events and randomly selected 153 out of 12 692 nonnoise events, were used in the five‐fold cross‐validation. Extraction of one intracardiac electrogram waveform was a manual process, and to avoid excessive effort, representative nonnoise events were selected by two electrophysiologists, instead of using all nonnoise events. The data set was divided into five sub‐data sets. The first data set was used for testing, the second was for validation, and the other three were for training. Next, the second data set was used for testing, the third was for validation, and the other three were for training. In this way, the same analysis was performed five times, with the same data set not selected for testing or validation. In addition, five‐fold cross‐validation with the weighted loss function was performed to reduce false negatives. RM data from Boston Scientific were analyzed in the same way (Figure 2). The confusion matrices and the Receiver Operating Characteristic (ROC) curves of each fold in each weighted loss function were analyzed.

### 2.4 Statistical analysis

Statistical optimization of the CNN was performed through iterative training using PyTorch. Once a final fitted model was obtained, the diagnostic performance was formally analyzed. Five‐fold cross‐validation was performed to predict noise events. The ROC curve and a weight loss function were employed to reduce false negatives. All analyses were performed in Python using scikit‐learn.

## 3 RESULTS

### 3.1 RM data

The 14 809 RM data points analyzed consisted of 5214 PM, 6333 ICD, 3173 CRTD, and 89 CRTP data (Table 1). Of these, 12 925 and 1884 RM data points were from Medtronic and Boston Scientific, respectively.

**TABLE 1:** RM data.

	PM	ICD	CRTD	CRTP	Total
Medtronic	4669	5314	2871	71	12 925
Boston Scientific	545	1019	302	18	1884
Total	5214	6333	3173	89	14 809

Abbreviations: CRTD cardiac resynchronization therapy defibrillator; CRTP cardiac resynchronization therapy; ICD implantable cardioverter defibrillator; PM, pacemaker; RM, remote monitoring.

The causes of noise events were lead failure, magnetic interference, or unknown.

### 3.2 Five‐fold cross‐validation

**FIGURE 3:**
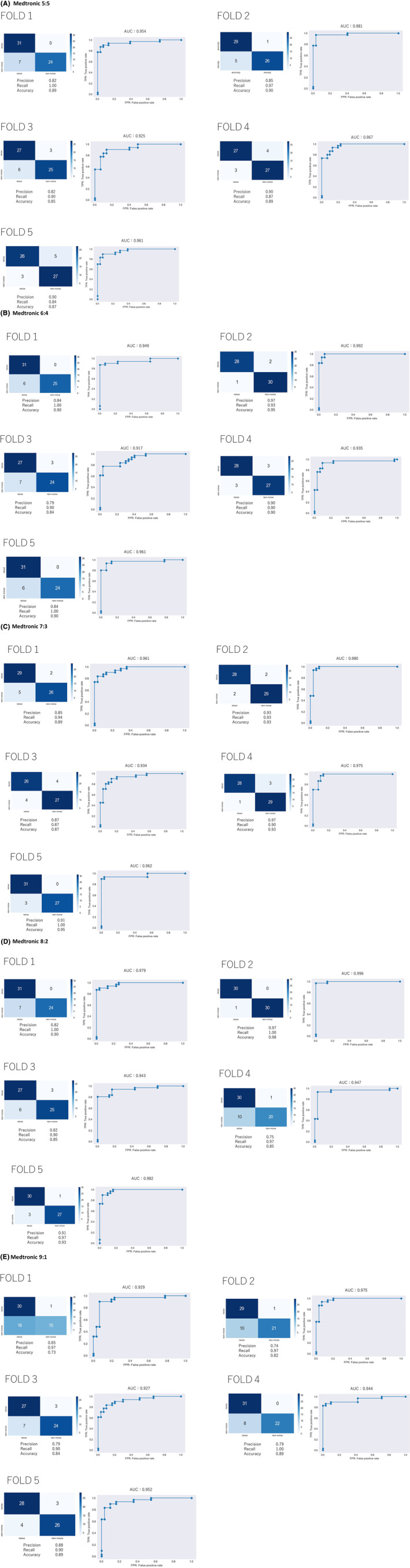
The confusion matrixes and receiver operating characteristic (ROC) curves of all five‐folds in each weighted loss function in Medtronic data. Weighted loss function (A) 5:5, (B) 6:4, (C) 7:3, (D) 8:2, (E) 9:1. AUC, the area under the curve.

**FIGURE 4:**
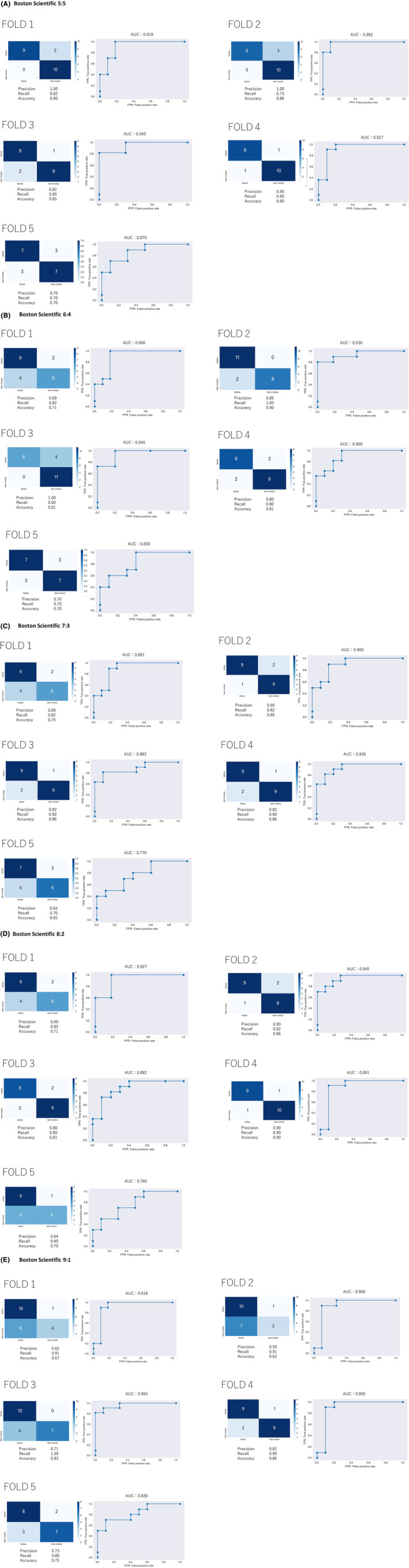
The confusion matrixes and receiver operating characteristic (ROC) curves of all five‐folds in each weighted loss function in Boston Scientific data. Weighted loss function (A) 5:5, (B) 6:4, (C) 7:3, (D) 8:2, (E) 9:1. AUC, the area under the curve.

The precision rate (positive predictive value), recall rate (sensitivity), F1 score, accuracy rate, and the area under the curve were respectively 85.8 ± 4.0%, 91.6 ± 6.7%, 88.4 ± 2.0%, 88.0 ± 2.0% and 0.958 ± 0.021 in Medtronic data (Table 2; Figure 3A) and 88.4 ± 12.8%, 81.0 ± 9.3%, 84.1 ± 8.3%, 84.2 ± 8.3% and 0.928 ± 0.041 in Boston Scientific data (Table 3; Figure 4A).

**TABLE 2:** The average of five‐fold cross‐validation in Medtronic.

Noise:Nonnoise	Average of five‐folds				
	Precision rate (positive predictive value)	Recall rate (sensitivity)	F1 score	Accuracy rate	AUC
5:5	85.8 ± 4.0%	91.6 ± 6.7%	88.4 ± 2.0%	88.0 ± 2.0%	0.958 ± 0.021
6:4	86.8 ± 6.9%	94.6 ± 5.1%	90.3 ± 3.9%	89.8 ± 3.9%	0.951 ± 0.028
7:3	90.6 ± 4.8%	92.8 ± 4.9%	91.6 ± 3.4%	91.4 ± 3.3%	0.962 ± 0.018
8:2	85.4 ± 8.6%	96.8 ± 4.1%	90.6 ± 5.7%	90.2 ± 5.5%	0.969 ± 0.023
9:1	77.0 ± 8.4%	94.8 ± 4.5%	84.6 ± 4.4%	83.4 ± 6.6%	0.945 ± 0.020

*Note*: Data: mean ± standard deviation.

Abbreviation: AUC, area under the curve.

**TABLE 3:** The average of five‐fold cross‐validation in Boston Scientific.

Noise:Nonnoise	Average of five‐folds				
	Precision rate (positive predictive value)	Recall rate (sensitivity)	F1 score	Accuracy rate	AUC
5:5	88.4 ± 12.8%	81.0 ± 9.3%	84.1 ± 8.3%	84.2 ± 8.3%	0.928 ± 0.041
6:4	80.8 ± 12.7%	78.4 ± 14.9%	78.4 ± 8.3%	78.6 ± 8.3%	0.902 ± 0.045
7:3	77.4 ± 10.6%	82.8 ± 8.2%	79.8 ± 8.7%	79.6 ± 8.7%	0.876 ± 0.063
8:2	78.6 ± 11.9%	84.8 ± 4.8%	81.1 ± 6.7%	79.6 ± 8.9%	0.881 ± 0.072
9:1	69.4 ± 9.2%	90.4 ± 7.1%	78.1 ± 6.1%	74.6 ± 10.2%	0.902 ± 0.048

*Note*: Data: mean ± standard deviation.

Abbreviation: AUC, area under the curve.

### 3.3 Five‐fold cross‐validation with weighted loss function

Clinically, false negatives are crucial because missed events may be followed by catastrophic adverse events, such as near syncope, syncope, and sudden death. Subsequently, an analysis with a weighted loss function was performed to increase the weights of the noise events.

For Medtronic or Boston Scientific events, the larger the weight of the noise event, the greater the recall rate (Tables 2 and 3). The confusion matrixes and the ROC curves in each fold with weighted loss function were shown in Figures 3B–E and 4B–E. However, weighted loss function could not achieve the recall rate of 100%.

### 3.4 Gradient‐weighted class activation mapping (GradCam)

**FIGURE 5:**
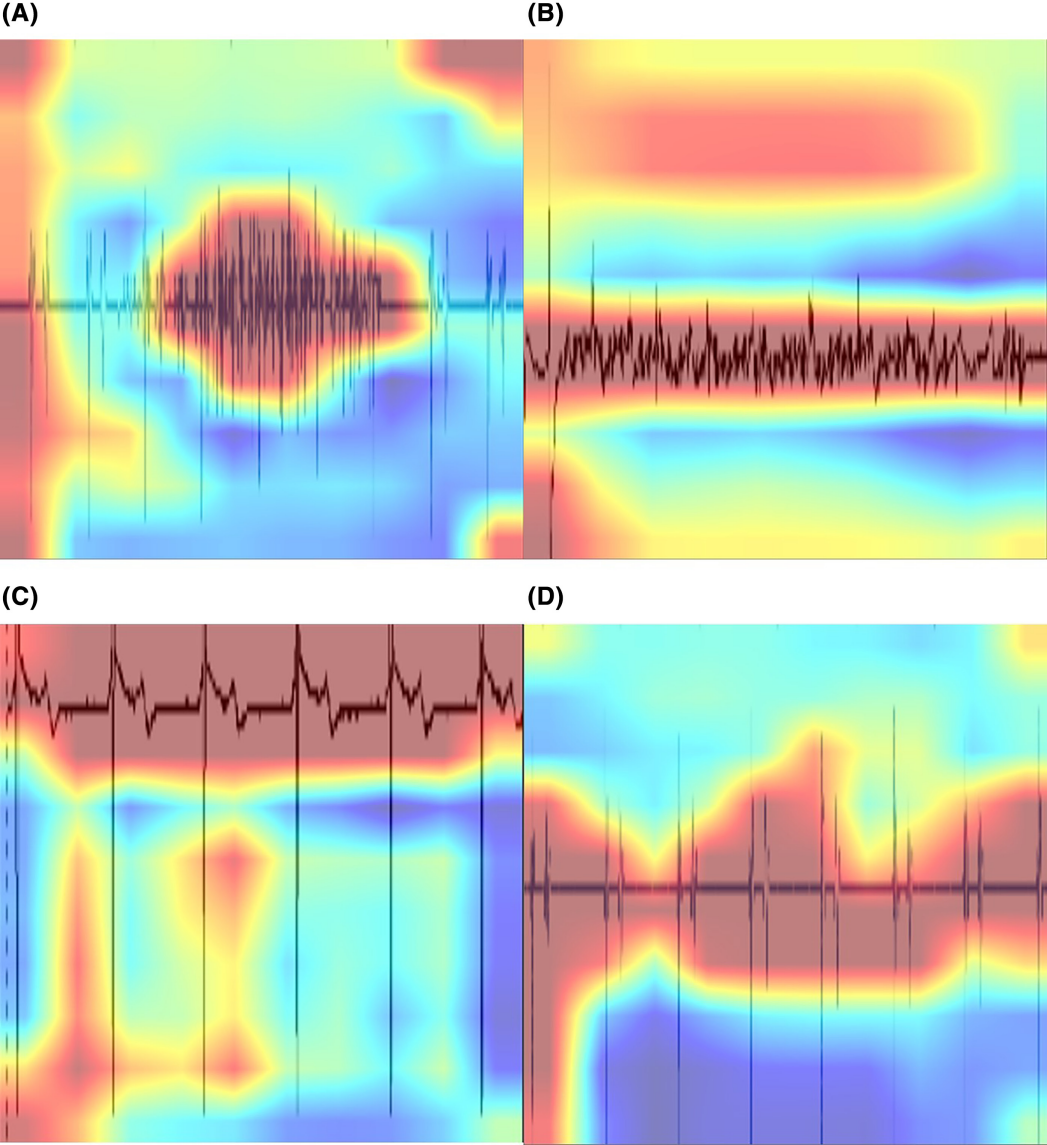
Gradient‐weighted class activation mapping (GradCam). Red color indicates the most focused area by artificial intelligence (AI). AI focused on the noise area of the intracardiac electrogram in noise events (A, B) and the overall intracardiac electrogram in nonnoise events (C, D).

GradCam is a method used to determine the part of the intracardiac electrogram focused on by AI.^23^ In noise and nonnoise events, the AI focused on noise and the intracardiac electrogram, respectively (Figure 5).

## 4 DISCUSSION

### 4.1 New findings

The main finding of this study is that AI can accurately distinguish noise events by learning the RM data from each manufacturer, even though the intracardiac electrogram was just picture, but not digital data. With the additional weight loss function, the false negatives decreased. This is the first report of AI analysis using RM data.

### 4.2 Importance of capturing noise events early

The causes of noise events were lead failure, EMI, loose set screws, and myopotential. Lead failure has resulted in life‐threatening events, especially in patients with cardiac pacing dependence, clinical lethal arrhythmia, and high‐voltage ICD leads.^6^, ^7^, ^8^, ^9^, ^10^ RM can detect lead failure earlier,^11^, ^12^, ^13^ which may result in the reduction of inappropriate ICD shocks.^14^, ^15^ However, lead failure is often noted only by arrhythmic events and not by impedance abnormalities.^14^, ^16^ In such cases, human analysis of intracardiac electrograms of arrhythmic events is needed to identify lead failure. In our previous study, only 32 (76.2%) of 42 lead failure events were detected as only arrhythmic events.^17^ EMI, loose set screws, or myopotential^24^, ^25^, ^26^ can also lead to life‐threatening events, especially in patients with cardiac pacing dependence, clinical lethal arrhythmia, and ICDs. Therefore, early detection of noise events is very important.

### 4.3 Huge workload of human analysis of all arrhythmic events

Time to event detection was longer for the atrial lead than for the ICD lead because recent CIEDs lack the function to detect noise events in the atrial lead port earlier. Therefore, to detect noise events in the atrial lead port earlier, precise analysis of atrial arrhythmias is necessary. However, among the transmitted data in patients with CIEDs, atrial arrhythmic events were the most frequent.^27^, ^28^ In a worldwide Home Monitoring database analysis,^27^ atrial arrhythmias were responsible for more than 60% of alerts in PMs and CRTDs and for nearly 10% of alerts in dual‐chamber ICDs. Analysis of all atrial arrhythmic events with intracardiac electrograms was very time‐consuming and had very low specificity for the detection of noise events. In contrast, noise events in the ICD lead port were frequently detected by alert events, such as ventricular fibrillation events, lead integrity alert events, and impedance abnormalities. However, not only impedance abnormalities, but analysis of ventricular arrhythmic events was necessary to detect lead failure earlier, because the proportion of lead failures detected by arrhythmic events was significantly higher than that detected by impedance abnormalities.^17^

### 4.4 AI analysis

Artificial intelligence and ML in medicine are currently areas of intense exploration, showing the potential to automate human tasks and even perform tasks beyond human capabilities. For example, AI can predict atrial fibrillation,^19^ hypertrophic cardiomyopathy,^29^ left ventricular dysfunction,^20^, ^21^ response to CRT,^30^ serum potassium level,^31^ gender and age,^32^ and 1‐year mortality^33^ from electrocardiograms of sinus rhythm. However, an analysis of the intracardiac electrogram has not yet been reported. This study showed that the AI algorithm could predict noise events in intracardiac electrograms with high diagnostic performance, especially with a weight loss function. In the Boston Scientific data, the recall rate or accuracy rate in five‐fold cross‐validation with the weight loss function was relatively low, which might have been caused by the small number of events.

If the trained AI is used in clinical situations, no false negatives are required because missed data may sometimes lead to catastrophic adverse events, such as near syncope, syncope, or sudden death. However, even though a weighted loss function was employed, it was difficult to achieve no false negatives. There are several reasons for this finding. The first was the small number of noise events because they were rare. Second, the intracardiac electrogram did not contain digital data, but just a figure or picture. This may be a disadvantage of AI analysis. Recently, digital data from intracardiac electrograms have become available. If digital data are used for AI, no false negatives might be achieved.

In the future, it is expected that AI can precisely diagnose arrhythmic events. For example, even though the arrhythmic event was diagnosed as ventricular arrhythmia by CIED, it was frequently diagnosed as supraventricular arrhythmic events by human analysis. Arrhythmic diagnosis by CIED is frequently incorrect, which may lead to an increased workload for RM data analysis. If AI could precisely diagnose arrhythmic events, the workload for RM data analysis would decrease.

### 4.5 Limitations

Several limitations of this study must be considered. First, in some company events, it was difficult to extract only an intracardiac electrogram. Thus, it was impossible to analyze all RM data. Second, even though the number of RM data points in this multicenter study was large, the number of noise events might have been too small for precise AI analysis because the noise events were rare. Third, the expression of intracardiac electrograms was too different among the companies to analyze the noise events from multiple manufacturers simultaneously. Therefore, an analysis of each manufacturer was necessary. Fourth, instead of analyzing all nonnoise events, representative noise events were selected by an electrophysiologist. If all nonnoise events had been used for the analysis, the recall or accuracy rate would have been higher. However, the representative data were selected by an expert electrophysiologist; therefore, this analysis seemed to be acceptable, and a high recall or accuracy rate was achieved. Fifth, the analyzed data were just image, but not digital data, because it was impossible to pull out digital data from previous remote monitoring data. If rule‐based algorithms, for example, “detection of the cycle length of less than 100 ms” were employed in digital data, the sensitivity would dramatically increase. Seventh, the analyzed data have vertical variability because of the original electrocardiogram position, but not additional change or augmentation, which may influence the AI analysis. However, the GradCam could strongly focus on the electrocardiogram, which might not be influenced by the vertical position of intracardiac electrogram.

### 4.6 Conclusions

The trained AI algorithm could predict noise events in intracardiac electrograms with high diagnostic performance, especially with a weight loss function. This model requires further refinement and external validation, but it may hold promise for the early and accurate detection of noise events in RM data.

## CONFLICT OF INTEREST STATEMENT

Nobuhiro Nishii and Hiroshi Morita belong to the Endowed Department of Medtronic Japan Co. Ltd. Nobuhiro Nishi received lecture fees from Medtronic Japan Co. Ltd. and Boston Scientific Japan. None of the other authors have any additional relationships with industry.

## ETHICS STATEMENT

The study protocol was approved by the Institutional Review Board and/or Medical Ethics Committee of each hospital.
